# Community Transport’s Dual Role as a Transport and a Social Scheme: Implications for Policy

**DOI:** 10.3390/ijerph21040422

**Published:** 2024-03-30

**Authors:** Léa Ravensbergen, Tim Schwanen

**Affiliations:** 1 Transport Studies Unit, School of Geography and the Environment, University of Oxford, South Parks Road, Oxford OX1 3QY, UK; tim.schwanen@ouce.ox.ac.uk; 2 School of Earth, Environment & Society, Faculty of Science, McMaster University, 1280 Main Street West, Hamilton, ON L8S 4K1, Canada

**Keywords:** community transport, older adults, transport scheme, social scheme, policy silos, interviews

## Abstract

Community transport comprises diverse local, not-for-profit, and primarily volunteer-run transport schemes that operate across the United Kingdom. These schemes support the travel needs of thousands of people, most of whom are older, live in rural areas, and have few other transport options. Further, this transport sector is unique in that most schemes are designed, created, and run by older people themselves. And yet, community transport has thus far received relatively little attention in both policy and research. Using semi-structured interviews with community transport providers in Oxfordshire, this paper proposes community transport as a practice guided by phronesis and argues that it has been made to hold a dual role as both a transport and a social scheme. The transport it provides is unique in being made low-cost, flexible, and functionally accessible. It has also been made into a social scheme as it helps those with few other options, provides benefits that extend beyond the transport realm, and fosters community. Though this dual role means that community transport has many cross-sectoral benefits, this type of service provision is found to be overlooked in both national and local policy, which has enabled the constitutive role of phronesis in community transport. Given this, there are challenges ahead for the sector in both ensuring its sustainability and maintaining its ability to respond closely to users’ needs.

## 1. Introduction 

Across the United Kingdom, a large network of community transport schemes helps thousands of people meet their essential travel needs. In 2012, 1900 community transport organizations operated in England alone [1]. A wide range of schemes constitute community transport, including dial-a-ride services, volunteer car schemes, and community buses. Though diverse in the service they provide, all schemes share the aim of meeting local transport needs that are not met by conventional transport, such as commercial or public sector transport. Community transport also differs from conventional transport in that it follows a not-for-profit and community-based model and in most cases is volunteer-run and managed [2]. Most volunteers are older people themselves who are responding to the need for transportation in their communities. In 2020–2021, community transport schemes delivered over 70,000 passenger trips, typically to people who not only live rurally but are also older, isolated, or have a disability [3]. And yet, this form of transport has received little attention to date in both academia and practice. 

This paper responds to this gap in the literature by considering what community transport provides to its users. Our findings indicate that community transport has come to hold a dual role as a transport and a social scheme with many cross-sectoral benefits. The transport its volunteers provide is unique in being made low-cost, flexible, and functionally accessible. Community transport has also taken on roles of a social scheme, in that it has been made to meet users’ broader social needs, to help foster community, and to reduce isolation. The paper concludes by positioning the community transport sector within the UK and international policy context. Though community transport offers services that could fall under many different policy jurisdictions, no government department or level of government has taken responsibility for these schemes in the UK. This lack of recognition in the policy landscape raises important questions given the important service community transport provides to people at risk of experiencing transport poverty, the combination of social and transport disadvantage [4]. 

## 2. Context

The first community transport service in the UK began in the early 1960s, but this kind of provision grew in prominence in the 1980s, as did research exploring and mapping out the service [5,6,7,8]. Over the past 20 years, few papers have considered community transport. Those that do also describe community transport’s diverse and beneficial schemes [9] and highlight how community transport organizations are underpinned by social capital [10,11]. Gray, Shaw, and Farrington [10] argue that strong social capital is important in conferring mobility through community transport, especially amongst certain demographics including those without access to a car. More recently, Hagan [11] showed the ways in which community transport provides more than transportation: it provides a “third space” for building relationships, reduces isolation, and helps people maintain autonomy. 

Other research has considered community transport indirectly. This work instead focuses on the potential for flexible or demand-responsive transport options in improving social inclusion in areas that are considered difficult to cover by public transport, notably rural areas of the UK [12,13,14]. Because customers can book door-to-door lifts in advance, community transport is presented as an existing flexible and/or demand-responsive option in these studies. Interestingly, community transport is not always presented as a clear solution to rural transport. For instance, in their study based in rural areas of southwest England and Wales, Shergold and Parkhurst [13] note that some interviewees associated community transport in negative ways with being for “old” and “less able” people. In sum, community transport has received little direct attention in research to date, especially in recent years. The two recent papers that consider community transport tend to focus on the role of social capital in its delivery of transport. 

In an international context, similar services to those in the UK can be found in Australia. Across the country, community transport schemes provide low-cost lifts to those with few other options. Annually, community transport in Australia serves approximately 238,000 clients and makes 5.5 million trips [15]. Unlike the UK, it seems the Australian schemes are more restrictive in who they serve [16]. Further, a large proportion of this sector’s funding is secured through the Commonwealth Home Support Programme (CHSP), which provides transport to eligible seniors [15]. Of note, this may not be the case for long as changes have been proposed to this model [17]. Two recent papers focusing on community transport in the Australian context have examined the potential for community transport to be provided through a Mobility as a Service (MaaS) model. Focusing on providers, one paper found that community transport operators were excited about this model [18]. The other paper examined users’ willingness to pay for community transport if it were provided through a MaaS model. Results indicate that users are willing to pay less than the cost of providing the service [17]. 

Beyond Australia, community transport schemes most closely resemble paratransit systems in both developing economies and in the United States and Canada. In Asian, South American, and African contexts, paratransit generally refers to informal and entrepreneurial transport schemes that respond to gaps in more formalized public transport [19,20,21,22]. While these schemes vary immensely, they are characterized by flexibility and informal governance [19,22], as is community transport. Unlike community transport, informal paratransit does not explicitly target older people or people with disabilities. In the United States and Canada, paratransit refers to flexible and individualized transport schemes that complement fixed route transit and target older adults or people with disabilities. While community transport shares these characteristics, these paratransit schemes tend to be more formalized, as they originate from legislation that prohibits discrimination based on disability, including for transportation (e.g., the American with Disabilities Act (ADA) of 1990) [23,24]. Taken together, community transport shares the flexible aspects of more informal paratransit services (common in developing economies), and the mobility demands of more formalized paratransit services (common in places with disability legislation). 

The current paper considers what UK community transport provides to its users. In doing so, it builds on the small and somewhat dated literature (with one recent exception: Hagan [11]) on community transport and the burgeoning international literature on flexible paratransit schemes. 

## 3. Methodology 

### 3.1. Theoretical Framework 

This study follows the tradition in transport studies of considering mobility as emerging from practices, or routinized ways of doing things [25,26]. This paper draws on practice theory broadly by considering how community transport is produced through a nexus of body/knowledge/things complexes that can become habitual but that nonetheless require deliberate effort [27]. As such, the mobility community transport provides is not framed as intrinsic, nor rigidly set, but as a practice that is stabilized to a degree. 

Further, the paper posits the practice of community transport as grounded in phronesis. Phronesis, or practical wisdom, refers to how to reasonably address and act on social problems in a particular context [28], or what Tyfield [29] calls “situated strategic, ethical wisdom” (p. 345). Phronesis differs from other forms of wisdom, including episteme, techne, nous, and sophia (Table 1). 

While similar to common sense, phronesis has an ethical element to it: phronesis focuses on “good” rather than “correct” action. As such, it involves knowing what is the appropriate, or best, thing to do in a given circumstance [30]. Following Burbules [31], phronesis as a virtue and phronesis as a practice are closely related, as ethics are a form of practice. People learn phronesis through engaging in practices, and the learning of a practice is a phronetic endeavor [31]. In considering community transport as a practice grounded in phronesis, this paper responds to calls for social scientists to embrace phronesis [28], and mobilities research to shift toward phronesis to understand the societal challenges faced today [29]. Further, focusing on phronesis emphasizes a different way one can run a transport service: one designed by and for older people themselves that is guided by practical wisdom rather than profit. 

### 3.2. Methods 

This study is based on qualitative fieldwork that took place between May and September 2022 in Oxfordshire, a county in the southeast of England. Oxfordshire is locally governed by Oxfordshire County Council and consists of five districts: City of Oxford, Cherwell, South Oxfordshire, Vale of White Horse, and West Oxfordshire. The county comprises many villages and towns and has only one small city with approximately 150,000 inhabitants: the City of Oxford. Outside of the City of Oxford, car travel and rural bus routes comprise the main form of conventional transport in the county, though the bus service has been shrinking over the past 15 years due to cuts in bus subsidies [32]. 

Given both the lack of literature on community transport and the uniqueness of these schemes, schemes that are designed, managed, and run for and by older adults, a goal of the study was to understand what community transport provides to its users. A quantitative approach, such as a survey, was deemed inappropriate. This decision was made because surveys require the researchers to generate pre-set categories of service provision. The goal was instead to let providers describe in their own words what these schemes do. Further, a quantitative approach would not capture how these schemes came to operate as they do today. Therefore, interviews as a qualitative method were chosen to capture the nuanced and varied service that community transport provides [33].

A list of all community transport organizations in operation in the county was compiled by the Oxfordshire County Council in a directory [34]. All 80 organizations that operate across Oxfordshire and have an email address were contacted and invited to take part in a semi-structured interview. Then, six schemes that did not have email addresses were contacted by phone and invited to take part. Participants were invited to complete the interviews in-person or remotely (i.e., by phone or using an online platform). Interview questions focused on organizational structure, procedures, benefits, and challenges. All required ethical reviews were approved by the university before the project began. 

A total of 28 interviews were completed with community transport providers at 21 different schemes. In other words, in some cases two providers from the same scheme were interviewed. These interviews ranged from 20 to 90 minutes in length. Most interviews were approximately 50 minutes. Interviews were conducted by the first author, who presented herself as an expert in transportation but not necessarily one in community transport. Her Canadian accent also made clear that she was not from the UK. Given the interviewer’s lack of expertise on these specific schemes and her “outsider” positionality, most interviewees easily and quickly understood their role as explaining these schemes to her. This was beneficial given that the goal of this study was to understand how community transport has been made to provide the service it does. 

Interviews were transcribed verbatim and analyzed using thematic analysis [35,36]. The thematic analysis method begins with data familiarization. In this case, the lead author began identifying and jotting down preliminary themes as they transcribed the interviews. Throughout this process, initial codes were developed and refined into potential themes. Through sharing, discussing, and reviewing these codes and themes with the second author, final themes were identified and defined. The first author then systematically read through all transcripts in NVivo 12, coding all relevant sections into the identified themes. This process was reflexive, meaning that the researchers were actively engaged in the data and sought to understand how it was produced at the intersection of the researcher-researched interaction [35,36]. This paper focuses on those themes that describe and explain how community transport have been made to operate from the providers’ perspective.

## 4. Findings and Discussion 

Community transport schemes in Oxfordshire all operate in unique ways, but two major types of schemes can be identified: community minibuses and volunteer car schemes (VCS). These two types of schemes run in either urban (City of Oxford), town, or village settings, though some operate across the county, or parts of the county. Table 2 displays the number of each type of scheme included in this study corresponding to its geographical categorization. Mirroring community transport schemes across the country and county, most schemes included in this study operated in non-urban areas (Table 2). 

VCSs provide rides to individuals in the driver’s personal vehicle. Passengers must book ahead and often pay a mileage fee. Most schemes restrict the destinations eligible, with health-related appointments being the most popular. These schemes tend to be small-scale, volunteer-run, and run most successfully in small villages, potentially owing to the importance of social capital in the effective operation of these schemes [10,11]. Community minibuses run on one of two permits. First, minibuses running on Section 19 of the Transport Act 1986 permits carry of restricted groups of passengers (e.g., registered members taking part in specified community activities) on a pre-booked flexible route [37]. Second, minibuses running on Section 22 permits run much like commercial operators in that they provide a fixed-route, local bus service that can be used by the public (and not just registered members). However, the minibuses cannot compete with commercial operators; their goal is to provide transport where no commercial route exists [37]. Table 3 displays the key characteristics common of these schemes, showing how VCSs and Section 19 minibuses have similar operations but different vehicles, while Section 19 and Section 22 minibuses have similar vehicles but different operations. 

Throughout the interviews, it emerged that community transport has been made to be both a transport scheme and a social scheme. Table 4 highlights the key characteristics of this dual role community transport holds, and the following sub-sections detail how these schemes have come to be this way. 

### 4.1. Community Transport as a Transport Scheme 

Both community minibuses and VCSs offer a unique transport service that, when compared to conventional transport options, is made more low-cost, flexible, and functionally accessible for passengers. 

#### 4.1.1. Low-Cost Transport: “They Don’t Actually Pay Anything”

This paper follows Plyushteva [38] conceptualization of transport (un)affordability as a process. In other words, it examines how mobility is afforded through context-specific transactions, journeys, and budgets of transport users. Because the perspective of the user was not directly captured in this study, the focus is on community transport schemes’ fares, as constructed by its volunteers. These volunteers make community transport fares as low-cost as possible. Of course, keeping fares as low as possible does not necessarily imply that users find these schemes affordable. However, through its not-for-profit model, schemes do not aim to make a profit and volunteers deliberately find ways to keep fares low. These low-cost fare structures have their basis in phronesis, or in volunteers aiming to make their schemes more affordable because they recognize this will help their passengers, many of whom live on (very) low incomes, access community transport. The strategies to make fares low-cost have been routinized by volunteers and differ for VCS and community minibuses, both of which are described below. 

Most VCS passengers are asked to pay a mileage fee, which is given directly to the drivers. The fare is trip-specific and is set to offset the cost of the trip (petrol, vehicle maintenance) but not the labor required or the cost of the vehicle (both of which are provided by the volunteer). Many interviewees noted how low the mileage fee was set, especially when compared to other options, such as a taxi. For instance, a South Oxfordshire volunteer driver explained: “*we only charge a mileage charge […] we will take them. We will wait for them. And then bring them back. And that comes to £17.00. […] It’s 36 pounds. One way. For a cab*”. Further, some schemes do not charge a mileage fee but instead ask for a donation. Other organizations even refuse donations, meaning volunteers not only provide their time and their vehicle but pay for running costs out of their own pocket. 

VCSs can offer a low-cost service because providers keep overheads as low as possible. Most organizations do not need a dedicated space out of which they work, with coordinators taking calls on either their personal phone or a phone paid for by the organizations. Costs therefore include minimal phone bills and occasional printing to advertise the service. For instance, a volunteer for a scheme in West Oxfordshire shared that “*we’ve got no overhead, so we are quite cheap, really*”.

There are some exceptions, i.e., a handful of larger VCSs that require an office and pay (some) staff. For instance, a VCS operating in a town explained how they pay minimal rent, their telephone costs, and part-time staff through funds from the County Council and donations:

*Oxford County Council do [fund the scheme], and our clients do. We ask our clients every year to contribute. We send out a letter asking them, would they contribute to the running of the office […] The money that we asked for, it’s usually a small donation, suggested £5 or £6 (laughs). And they’re all very happy to give that.*

Even in this case of a large VCS with higher overhead costs, the costs passengers contribute are minimal given the low overhead of the organization, which is paid for through funds acquired through the County Council. Taken together, regardless of VCS size, community transport providers make the fares charged low. In all cases, this is done by using volunteers’ personal vehicles and not charging the costs of the labor required. In some cases, this also requires volunteers paying out-of-pocket costs to offset the trip (e.g., for fuel and maintenance), to perform administrative tasks, or to secure funding from grants. Making these fares low may be routine but still requires effort from committed volunteers. 

Community minibuses are also kept low-cost, though the strategies undertaken to do so differ from VCS. All community minibuses charge fares. However, policies have been put in place so that they can accept concessionary bus passes, a national scheme that provides free bus travel to older adults and people with disabilities. This means that passengers with these passes can use the minibus for free, while the community transport scheme can claim their fares back from local government. As a minibus operating in the Cotswolds stated: “*For the 95% of our passengers, they have a bus pass, a concessionary bus pass, so they don’t actually pay anything*”.

Section 22 minibuses are open to the public, and those who do not have concessionary bus passes must pay a fare. Interviewees, such as the Cotswolds driver above, shared how: “*We charge fares, which are very low, but we do charge fares*”. As was the case with VCSs, these fares are kept as low as possible by providing the driver labor through volunteers. Section 19 minibuses also claim bus pass fares from local government, but all passengers, including those with a concessionary bus pass, pay a (usually flat) membership fee because Section 19 permits are membership based. These fees are also kept low. For instance, a minibus operating in the City of Oxford (now closed) shared the following:

*[We] charge people an annual membership which is about £60 a year. And if they wanna go once a week they go once a week, if they wanna go five times a week, then they can go to five times a week. And then there’s no additional cost to that [because they can use their bus passes].*

In another cross-county minibus scheme, membership fees were as low as £3, and then passengers “*pay £1.50 an hour—and [they] can put ten of [their] friends on the bus*”. In yet another example, a Section 19 minibus provides three levels of membership, the most affordable of which costs nothing to join and the most expensive of which costs £45 per year. All in all, even when a membership is required, community minibuses schemes aim to provide affordable fares. 

As was the case for VCSs, bus pass fares, direct fares, and membership fees tend to cover the running costs of community minibuses (petrol, maintenance). Funding to cover the remaining costs, including staff (when not fully volunteer-run) and replacing buses must be raised elsewhere. Two organizations historically received funding from the County Council. One received funding from Section 106 Agreements (which require developers to set aside funds to contribute toward community infrastructure), and the other four rely on piecemeal funds from diverse places including grants, private fundraising, and donations from corporations, charities, trusts, and private citizens. As was the case for VCSs, the fares are kept low by committed volunteers who either provide their labor free of charge or devote ample effort to securing funding from various means. In both cases, this act of keeping fares low is based in phronesis, or in response to knowing that many passengers might not be able to afford the service if they had to pay its “true cost”—i.e., the price that providers would have to charge if all incurred costs were properly monetized and taken into account. 

#### 4.1.2. Flexible Transport: “We Pick People up from Their Door”

Both VCSs and community minibuses emerged as transport schemes that were made flexible in a phronetic manner, especially when compared to fixed-route buses that are limited to rigid spatiotemporal service provision. In operating a door-to-door service, VCSs provide very flexible transport. Further, most lifts are scheduled to coincide with the time of a health appointment, a deliberate choice amongst volunteers wishing to provide passengers with convenience (again, a practice based in phronesis). A West Oxfordshire volunteer driver highlighted this flexibility by comparing the service to the bus: “*public transport to Oxford, is ... it’s possible, but it’s not convenient*”. She later elaborated by sharing the following:

*Travel by bus with long intervals between them and necessity to take one or more buses each way in a journey is often just too challenging for those who are not in the best of health… This week I took a gentleman to his doctor for his second visit in one day. He said he was in so much pain he just couldn’t have borne going on the bus again.*

This interviewee demonstrates how flexible and convenient VCSs have been made compared to other options, in this case the bus. In this example, VCS volunteers respond through phronesis to the needs of passengers who often are “*not in the best of health*” or in too much “*pain*” to go “*on the bus again*” by offering flexible, and door-to-door, transport options that a conventional bus service could not easily provide. Some volunteers even mentioned making detours on the way to or from health appointments to run an errand, such as to post a letter or to pick up something at a petrol station, to help meet passengers’ needs. Volunteers can provide this flexible transport because of the lack of formal rules guiding this service—rules that can restrict bus services. 

While VCSs are more flexible than conventional transport regarding the route they take, spatially, they do have the drawback of needing to be booked in advance. This is the case because it takes time and logistical effort to secure a volunteer driver, as a VCS volunteer operating in a town explained:

*We only accept bookings in advance. We’ve had one of the local surgeries who tried to get us to take bookings there and then, […] we said: “We can’t do that, because we’ve got to get a driver.” I mean we haven’t got a driver standing by every day of the week.*

Because the service relies on volunteer drivers, it is not on demand. Most VCSs suggest that passengers request the service at least a week in advance, as last minute trips are harder to organize. Community transport still, however, emerged as being made flexible, especially when compared to other (relatively) low-cost options such as buses. 

Community minibuses have also been made to provide more flexible transport to passengers. Like VCSs, Section 19 minibuses also offer a door-to-door service that must be booked in advance. Even Section 22 minibuses that run a fixed route provide relatively flexible transport. For instance, volunteer drivers will stop in front of passengers’ homes. As a minibus operating in the City of Oxford shared: “*We pick people up from their door, so they don’t have to walk to the bus*”. They also design their route to best suit older adults’ needs by connecting homes to shopping and social outings. For instance, the City of Oxford minibus volunteer explained, “*We take them to the destination, which may not necessarily be on a direct bus route*”. Examples of places for which small detours are made include in front of supermarkets or health centers. In this way, community transport schemes, both VCS and minibuses, make the transport they provide as flexible as possible to respond to passengers’ needs. 

#### 4.1.3. Functionally Accessibility Transport: “An Arm Is Usually All That’s Needed” 

A distinction emerged during the interviewees between transport that is technically accessible, i.e., that follows official guidelines to accommodate people with disabilities, and that which is functionally accessible, i.e., which enables mobility for people with disabilities. Most minibus schemes could not officially accommodate wheelchairs, making them technically inaccessible (of note, three were able to officially accommodate wheelchairs). However, the volunteers deliberately made these schemes more functionally accessible than conventional transport. For instance, a minibus coordinator operating in a town in Southwest Oxfordshire shared the following:

*The medical center is there. Not on the road. You have to walk up that hill. […] But we go into the medical center. So we come off the main road. We drive in. We turn around. And we move—maneuver—in their carpark. We drop them outside the door. That’s one reason they use us […] because if they’re going to the medical center, there’s a likelihood they probably can’t walk very well.*

Here, the community bus was made to be functionally accessible by providing door-to-door access to the medical center. A regular bus may be able to accommodate wheelchairs and so be technically accessible. However, it would likely drop people off on the main road, leaving passengers to contend with the hill up to the medical center’s entrance. This would make the bus functionally inaccessible. While those in wheelchairs cannot use the community minibus, volunteers have found a practical solution to make the buses more accessible: those with varying levels of frailness find the minibus more functionally accessible than regular buses because it “*drop[s] them outside the door*” of the medical center. 

Approximately half of the VCSs included in this study accommodate wheelchairs, usually in the boot of the volunteers’ car. Doing so requires deliberate effort on behalf of the volunteers, including ample logistical work (i.e., knowing which volunteers have both cars that can accommodate wheelchairs and the ability to store them away). For instance, a VCS driver operating in a town shared: “*we take wheelchairs. I mean, some people their cars are not big enough. So, you know, on the list of the drivers it will say ‘no room for wheelchair’ or something like that. So, you know, we—those of us that can—do*”. There are constraints to offering this accessibility, and some VCSs specifically refuse to give lifts to people using wheelchairs to protect their drivers. For instance, a West Oxfordshire VCS volunteer coordinator shared:

*One of the restrictions is that you have to be able to get in and out of a car unaided because we haven’t got the facility for—obviously—for getting people in and out of a car or for a wheelchair. A lot of drivers say “I can’t take a wheelchair. I’ve got a bad back” you know “I couldn’t lift a wheelchair in and out of a car.”*

Half of the VCSs were not technically accessible because they cannot accommodate wheelchairs. However, these technically inaccessible schemes all mentioned the ways in which they made the schemes functionally accessible by providing passengers with physical assistance. Here, “*an arm is usually all that’s needed*” (a West Oxfordshire VCS volunteer coordinator) to help passengers into cars and into destinations. A South Oxfordshire VCS volunteer shared:

*“But to be honest, most of the people that we get would struggle to get on and off the bus. You know, whereas we can get a car close up to their house. Help them in. Help them out the other end. They would struggle to get a bus.”*

Taken together, some community transport schemes are fully accessible, but most are not technically accessible. However, when this is the case many community transport schemes, both community minibuses and VCSs, are made to be more functionally accessible than conventional transport, as a phronetic response to the passengers’ needs. This can be done through providing a flexible, door-to-door service, by informally accommodating mobility aids, or by providing physical help to those in need.

### 4.2. Community Transport as a Social Scheme 

Not only has community transport been made to provide low-cost, flexible, and functionally accessible transport services, but the impact of these schemes has also extended beyond the transport realm. Indeed, many interviewees identified the dual role community transport plays as a transport and a social scheme. For instance, a community minibus driver in the Cotswolds shared: “*I think we see ourselves not so much as a transport company, but as a social service*,” while another community minibus volunteer said: “*That’s kind of the benefit, the obvious one being the mobility, but the bigger community spirit would be a secondary one*” .

Elsewhere we have argued that community transport is a care-driven scheme whereby providers identify, take responsibility over, and respond to community members’ needs [39]. Building on this argument, the community’s need for transport is just one of a bigger conglomerate of needs that those using these schemes have. Community transport providers recognize and take responsibility for meeting these wider needs, and they do so through phronesis. In this way, community transport emerged as a social scheme, one that is holistic in that it provides more than transport, targets groups vulnerable to transport poverty, and builds community. In fact, considering community transport solely as a transport service greatly oversimplifies the role of these schemes in people’s lives, as well as the effort that providers commit to caring for their communities. 

#### 4.2.1. Holistic Schemes: “We’re Not Just Dropping People off”

Community transport emerged as holistic; it goes beyond getting people from point A to point B. For instance, VCSs not only provide a flexible door-to-door service, but they also (when needed) help passengers navigate mobility at their trips’ origins and destinations. Volunteers at most schemes discussed how they help passengers navigate complex hospital buildings, the most common destination for VCSs. For instance, a South Oxfordshire VCS volunteer driver shared: “*We also help people with taking them into the hospital or doctor’s or whatever, you know, providing a wheelchair […], take them in, that sort of thing*”. Another interviewee explained:

*Actually, the reality of these schemes is: (1) it’s not just about the transport. Quite often they go to hospitals. And what people really value—whether they’ve got money or not—is actually help to get to wherever they’re going, whichever [hospital] ward. That that’s the really important bit, the bit outside of the car as well.*

In doing so, community transport provides mobility from A *into* B, in this case from home and into “*whichever [hospital] ward*” they need to get to. Other volunteers discussed helping people not just into B but *out of* A. Though less common, a few even helped people prepare for their trips, such as the following VCS driver who shared: *We shouldn’t go into people’s homes. But sometimes, you know, people have been standing there with their dressing gown on (laughs), and I know they’ve got an important appointment, because I’ve taken them before, and I’ve helped them to get dressed*”. Here, the volunteer not only drove the passenger to their appointment but also helped them prepare to leave their home by assisting “*them to get dressed.”* This was done because they recognized that the passenger’s needs were greater than the trip itself. This type of action is guided by phronesis, as another volunteer put it: “*We’re not supposed to help them. […] But I will say that I have helped them in the past. I mean even—Why would I stand and watch somebody struggle to get in and out my car when I can actually help them?*”

These volunteers went above and beyond providing transport because it was the morally good thing to do, yet something a bus driver could not do. This is possible because flexibility remains in the ways in which community transport operates, something that does not exist amongst more formalized public transport that must follow specific regulations and standards. 

The service is also made to be holistic *between* A and B. Notably, VCS volunteers shared how they frequently provide emotional support to passengers, as a community minibus operator explained: “*they usually go in for quite a stressful situation. […] having somebody there is really important […] we’re not just dropping people off*”. Again, this practical and context-based response (providing emotional support to those in stressful situations) is an act that is based in phronesis and can take place owing to the lack of rigid and institutionalized rules among VCSs. 

Community minibuses also emerged as holistic, though less so than VCSs given their more rigid set of rules. Even amongst Section 22 buses that run a fixed route, volunteers explained how they provided more than a pickup and drop off service. For instance, a City of Oxford minibus volunteer shared: “*we will help them with their bags and their trolleys into the bus. Take them home. Then those people that need help taking the shopping into their homes: we will do that. So, you know, that’s something that the bus wouldn’t do*”. The volunteer shows their awareness that they provide a more holistic service than regular buses. Help with “*bags*”, “*trolleys*”, and “*shopping*” is something, in their words, that “*the bus wouldn’t do*”—and something a bus could not do due to its more formalized nature. 

#### 4.2.2. Community Transport as a Last Resort: “It’s a Lifeline. It Is Literally Their Only Option”

As a transport scheme, community transport enables people to access places and people. However, the scheme is unique in the type of people to which it provides transport, namely those with no (or few) other options, i.e., those who are vulnerable to transport poverty or combined social and transport disadvantage [4,40]. As an interviewee who works for an organization that supports community transport shared, “*for people who use community transport, I mean, they say this all the time. It’s a lifeline. It is literally their only option*”. The bulk of passengers comprise older adults who have given up driving their car. In this way, community transport is a last resort for those with few other means to access important destinations, notably healthcare resources and grocery shopping. 

This is explicitly the case for VCSs, as the service is not open to the public. Instead, passengers must meet certain eligibility criteria. Of note, many schemes explicitly frame themselves as a “last resort” service for those with no other options; for example a South Oxfordshire VCS volunteer shared how they “*take people if they can’t get their own transport, or haven’t, you know, aren’t able for some reason, or haven’t got family or friends*”. This criterion, however, is not formally or rigorously implemented. Instead, it is an informal guideline that is enacted through local social networks. For instance, when the South Oxfordshire volunteer was asked how they ensure all passengers are without other transport options, they replied, “*We don’t. It’s just on trust really. And I mean we know most of the people in the village*”. 

Though community minibuses are open to the public, they still serve a role as a “last resort” for those with few other options. In fact, the permits these minibuses use forbid them from competing with commercial operators, meaning they exist to provide a service where none exists. As a community minibus driver in the Cotswolds shared:

*“There are many people, many old people living in villages, which have no shops nowadays, and have no bus service because they’re too small to support a commercial service. And the people there are A) lonely and B) deprived in the sense that they, they find it difficult to access services, and get to supermarkets, and so on. To do their shopping. So that is our main aim. […] we are there to help people who have no other means of transport to get to towns, to get to services in towns, doctors, dentists, shops, and all that sort of thing.”*

Though this minibus is publicly available, it still serves as a last resort for those “*old people*” who do not drive. Community transport, then, is made into a mechanism to help those who have “*no other means of transport*” to “*access services*”.

#### 4.2.3. Building Community: “A Social Club on Wheels”

Community transport emerged as a site that provides a space to connect with the community and to build relationships. For instance, a volunteer for a minibus operating in a town in the Southwest Oxfordshire commented on how passengers “*all know each other. They all know where everybody lives. They talk to each other*” and stated that sometimes “*people actually get on the bus for no other reason than that they can sit and talk to people*”. A community minibus coordinator even called their organization “*a social club on wheels*”. VCSs are also a place to connect with someone in the community (the driver) and to build a relationship. This can involve comforting people who are worried about their hospital appointment, as one VCS volunteer shared:

*Taxis are great. But if somebody’s having a bit of a wobble or a bit of an emotional wobble, they’ve been to get some tests or something, they’ve had to go by themselves. A taxi literally, the best ones in the world, picks them up, takes them home dumps them and goes. There is no “Are you okay? Are you going to ring your son about this?”, there’s no interaction.*

These examples show how these schemes are made to differ from conventional transport, such as taxis (“*even the best ones*”), as relationships form between the passengers (as was the case for the community minibus examples) and between the passenger and driver, through “*interaction*”.

Friendships can also be formed, as a South Oxfordshire VCS volunteer driver shared “it has a more personal connection, I suppose. […] For me it’s always interesting taking somebody from the village who’s probably been in the village longer than I have, and just chatting, as one does” while a West Oxfordshire VCS volunteer coordinator said: “And all our drivers do it, and not just because they like helping people, but because it’s fun. You meet interesting people. And have interesting chats and laughs and so on, on the way. And you make friends.” In this way, VCSs can break down isolation, as a South Oxfordshire volunteer shared:

*When you take them back and drop them off, a lot of them will say “oh, I’ve had such a nice time” because, you know, it’s the journey there and back, the chats. It’s all part of it. ‘Cause, you know, a lot of them are obviously either on their own or they don’t see very many people and, and to get to have somebody pick them up from their house and chat with them for the 40 min or whatever it takes.*

In this way these schemes build community, an intentional aspect of these schemes that is fostered by their volunteers who are practically responding to users’ need for connection. 

Volunteers can also informally check-in on people. For instance, a coordinator for a minibus operating in Southwest Oxfordshire shared: “*if somebody is booked for the bus, but they don’t turn up, we go and knock on their front door. And if nobody comes out, we will alert people*”. Another community minibus operator shared: “*if somebody is vulnerable then quite often I will reach out to the family […] it’s a small village. There will always be someone who knows the person*”. These are all phronetic practices—ethically good responses to situations—made possible by the informal and flexible rules that guide community transport schemes. 

## 5. Implications for Managerial Practice 

Community transport’s dual role as a transport and a social scheme results in many cross-sector benefits for those at risk of experiencing transport poverty and its many adverse impacts [4]. These benefits include accessing health services, meeting travel needs, reducing isolation, and building community. Given these, these schemes’ impacts could fall under the realm of at least three national government departments in the UK: the Department of Transport (DfT); the Department for Leveling Up, Housing and Communities (DLUHC); and the Department of Health and Social Care (DHSC). And yet, none manage community transport directly. 

First, community transport clearly falls within the remit of the Department for Transport (DfT), which supports the UK’s transport network and invests in transport infrastructure. The DfT is also responsible for providing transport-related policy, guidance, and funding to local authorities [41], all of which impact community transport. Of note, the DfT provides funding to community transport, though often indirectly [42]. While no statistics are compiled showing how community transport is funded, the DfT has supported it financially in various ways. For instance, the DfT has provided multiple large single-year funds support community transport (e.g., a £25 million Community Transport Minibus Fund in 2014); it provides £200,000 per year to the Community Transport Association (CTA), which supports schemes across the country; and its Bus Service Operator’s Grant (BSOG) pays some community transport operations [42]. 

While this indirect funding shows that the DfT does financially support community transport, the DfT frames the responsibility for funding community transport as a local issue. For instance, in a House of Commons Transport Committee Report, DfT representatives stated:

*Given their greater knowledge and experience of local transport issues, … it should be for local transport authorities, working in partnership with their communities, to identify the right solutions that meet the economic and environmental challenges faced in their areas and deliver the greatest benefits for their area. Local Authorities can use a variety of sources of finance, whether from central government or locally raised, to fund the provision of Community Transport [43].*

Therefore, while community transport is most clearly governed by the DfT, the DfT itself leaves “*local transport authorities*” and “*their communities*” to oversee, maintain, and identify sources of financial support for community transport. 

There are other sectors that work in areas clearly related to community transport but that do not currently seem to support these schemes in any way. The DLUHC aims to support local areas by fostering growth through creating jobs and delivering homes. To do so, the DLUHC supports community and faith groups and oversees local government, planning, and building safety [44]. As a locally run community group with a social aim, community transport clearly falls under the DLUHC’s mandate. Currently, however, community transport is not considered under this mandate. Further, in overseeing local government finance, the scale at which community transport is largely financed, and overseeing planning and land use, which contribute to community transport operations indirectly, the DLUHC should play a role in supporting community transport. And yet, community transport is not a consideration of the DLUHC. 

Finally, the DHSC governs and supports the country’s health and social care. It is responsible for setting current and future health-related policy, both domestically and internationally [45]. Community transport plays an important role in many people’s lives by helping them access healthcare. Most VCSs restrict the destinations they serve to healthcare, and many community minibuses include healthcare services as a destination. And yet, these schemes do not fall under the mandate of the DHSC. 

Locally, community transport is governed haphazardly. In 2004, a study noted how core funding for community transport from local authorities is vulnerable across the UK. Further, alternative sources of funding such as trusts, community funds, or other rural funding streams tend to provide financial support to generate new initiatives, rather than stable long-term financial support [9]. This is still the case today, as the Community Transport Association’s funding program resource guide lists a large patchwork of grants and funders to help organizations make ends meet [46]. This means that community transport exists on marginal funding, in Jones [9] words in “a state of financial crisis” where “their long-term future [is] almost always in doubt” (p. 141). Given the trend toward greater cuts in government spending in transport and elsewhere over the last 20 years [32], it is likely that this financial need has only worsened. 

In Oxfordshire, a relatively affluent UK county, the County Council does provide some financial support in the form of small start-up grants to help the development of new schemes, as well as some funding to train community minibus drivers [47]. Beyond that, the onus of managing these schemes is left to the providers themselves. Indeed, a “self-help toolkit” is provided to help communities “tackle local transport problems in a practical and effective way” ([47], paragraph 13), and if people require more assistance they are told to seek further support from a local a community development charity [47]. The County Council makes it clear that the responsibility of these schemes falls to community members themselves when it states on its website that community transport “shows what can be done when people take responsibility for solving their own problems” (paragraph 2). 

In sum, national policy overlooks community transport and downloads responsibility to local government, which downloads responsibility for these schemes to the providers themselves. It is important to note that despite this delegation of responsibility of community transport, there are many people working across these departments that care passionately about these schemes, something that became evident throughout the research process. The limited support from government nonetheless has implications for how community transport schemes operate. Indeed, this omission in the policy realm results in few formal guidelines and regulations for community transport as well as limited funding. These two conditions have ambiguous effects. On the one hand, they make community transport scarcer and leaner than its providers would like it to be, forcing many of the labor costs to be internalized by practitioners through volunteering. On the other hand, they also enable volunteers to shape community transport through phronesis, i.e., to make community transport a low-cost, flexible, functionally accessible scheme that transcends the realms of transport and social care. 

The case of community transport also highlights the issues with the siloed governance of transport, social services, and health in the United Kingdom. Community transport works across the mandates of all three departments. And yet, while the Department of Transportation plays a role in supporting and funding community transport, albeit disparately and indirectly, the other two departments have not engaged in community transport services. This paper therefore highlights the importance of different government departments communicating, collaborating, and working together to support community transport and the thousands of people it serves.

## 6. Discussion 

This study explores what community transport provides to its users and finds that it has been made to hold a dual role as a transport scheme and a social scheme. The transport it provides is unique in being low-cost, flexible, and functionally accessible. Community transport is also a social scheme that is holistic, as in it responds to more than just transport needs, alleviates transport poverty by helping those with few other transport options, and fosters community. It has been made this way through dedicated volunteers responding to users’ needs through phronesis, or practical wisdom. It can do so through its informal governance—the lack of strict rules and regulations allow for volunteers to make these schemes hold this dual role. Given how under-studied community transport is, with only a handful of recent studies on the topic in the UK (e.g., [9,10,11]) and Australia (e.g., [15,16,17]), the study responds to a gap in the literature. 

Community transport is a unique scheme that shares some aspects of informal paratransit found predominantly in developing economies and others of more formalized paratransit found in the United States and Canada. 

Because community transport is not formally incorporated into any level of government policy, its governance resembles more informal paratransit systems in developing economies [19,20]. In more formal Canadian and American schemes, users must meet (often stringent) eligibility criteria, undergo a standardized registration process, book trips in advanced through a formal system, and be driven by professional drivers [24]. As such, it is likely that there is less flexibility for providers to act through phronesis in the meeting of their passengers’ needs in these more formalized schemes. Clear regulations and standards exist guiding the services offered. These regulations and standards likely make it more difficult for a paratransit driver to, say, help passengers get dressed before their trips or to stop on the way home to run an errand. In this way, community transport is likely more adept at identifying local needs and responding to them than formalized paratransit. 

However, community transport’s informality comes with drawbacks, including a lack of structured funding or recognition in the policy landscape. As such, it is more vulnerable than more formalized paratransit systems in American and Canadian contexts. And this vulnerability is alarming. Indeed, two community transport schemes included in this study shut down during the research project. The formalization of transport services has, in the past, resulted in a loss of local and citizen-led contributions in innovations (e.g., see Truffer [48] on the professionalization of car sharing in Switzerland). Community transport may run a similar risk if it were to professionalize—the formalization of the service could result in the standardization of regulations and greater challenges for community members to phronetically respond to users’ needs. Questions remain of how community transport can formalize to ensure its sustainability without losing its flexibility. 

## 7. Conclusions

This study sheds light on a unique transport scheme that is designed, managed, and run by and for older adults and addresses transport poverty: community transport. Results indicate that community transport provides both a transport and social scheme with myriad cross-sector benefits. The transport it delivers is unique in being made low-cost, flexible, and functionally accessible. Further, it is a social scheme that has been made to meet passengers’ needs beyond transport, to provide mobility to those with few other options, and to build community. These unique aspects of community transport are often made possible through dedicated volunteers informally and flexibly responding to needs through practical wisdom. 

While this study responds to a gap in the literature by focusing on community transport, an under-studied transport scheme, future research is still needed. For instance, more research on community transport outside of Oxfordshire would be beneficial. Given that incomes in Oxfordshire are relatively high [49], work in other contexts is needed to establish whether findings of this study apply in other, and less wealthy, places. Further, a weakness of this study is that it has only focused on community transport providers. To assess the true impact of community transport, future work could complement this study by focusing on these schemes’ users (for instance, see [11]). 

Though its cross-sectoral benefits are clear, community transport is marginalized in the current policy landscape, both nationally and locally. Nationally, the DfT is the only department to govern community transport, and it downloads the responsibility for financing and governance to local authorities and the communities they serve. Local governments, on the other hand, provide no stable funding or support to community transport and download the responsibility to the providers themselves. This neglect in the policy landscape is what enables providers to shape community transport into a low-cost, flexible, and functionally accessible service that transcends the transport realm—the lack of formal guidelines and regulations creates a space for providers to do so through phronesis. While this flexibility has its benefits, its current marginalization in the policy landscape makes it vulnerable. Given the myriad social, mobility, and health benefits of these services to many of the most marginalized members of society, its lack of prioritization in the policy landscape is alarming. Moving forward, government sectors must take responsibility over community transport to ensure its sustainability—and doing so would require transcending the institutional silos of the DfT, DLUHC, and DHSC. 

## Figures and Tables

**Table 1:** Forms of Wisdom.

Phronesis	Episteme	Techne	Nous	Sophia
Practical wisdom	Scientific knowledge	Practical skill	Intuitive understanding	Deep wisdom

**Table 2:** Community Transport Schemes Included in the Study.

	City of Oxford (n = 2)	Town (n = 6)	Village (n = 11)	County/District Wide (n = 2)
Community bus (n = 7)	1	3	2	1
Volunteer car scheme (n = 14)	1	3	9	1

**Table 3:** Key Characteristics of Community Transport Schemes.

	VCS	Community Minibus	
		Section 19	Section 22
Capacity	1 person (usually)	Up to approximately 16 people	Up to approximately 16 people
Route	Pre-booked route, usually from passenger’s home to a health-related appointment	Pre-booked route, usually from passenger’s home to a pre-set destination	Fixed route, usually connecting residential areas with key services
Schedule	Flexible, based on appointment	Flexible, pre-booked	Fixed, often operates select dates (e.g., once per week) or times (e.g., mornings)
Ownership	Volunteer’s car	Minibus owned by community transport organization	Minibus owned by community transport organization
User cost	Mileage fee common	Membership fee	Bus fare concessionary bus passes accepted)
Passenger eligibility	Must have no other option (often)	Must be registered member	Open to public

**Table 4:** Community Transport as a Transport and as a Social Scheme.

Transport Scheme Characteristics	Social Scheme Characteristics
Low cost	Holistic
Flexible	Targets many few other options
Functionally accessible	Builds community

## Data Availability

The data presented in this study are available on request from the corresponding author. The data are not publicly available due to ethical concerns to maintain participant anonymity.
